# In the era of broad spectrum antibiotics, is ampicillin still an option?

**DOI:** 10.1186/1471-2334-13-S1-O14

**Published:** 2013-12-16

**Authors:** Cristina Popescu, Gabriel-Adrian Popescu, Alina Lobodan, Raluca Dulama, Doina Niculescu, Diana Tănase, Mihaela Rădulescu, Violeta Molagic, Cătălin Tilişcan, Liliana Ion, Mirela Cernat, Mirela Dinu, Iulia Caragea, Angelica Teniță, Georgiana Juganaru, Elisabeta Benea, Victoria Aramă

**Affiliations:** 1 National Institute for Infectious Diseases “Prof. Dr. Matei Balş”, Bucharest, Romania; 2 Carol Davila University of Medicine and Pharmacy, Bucharest, Romania

## Background

In the era of broad spectrum antibiotics it is sometimes difficult to choose the best antimicrobial regimens. Because of misuse and abuse of antimicrobial usage, the level of resistance is increasing and sometimes we do not have treatment options. Infectious diseases specialists traditionally have the leadership role in optimal use of antimicrobials. Antimicrobial stewardship represents a worldwide accepted concept in order to preserve currently available antibiotics.

## Case report

We present 4 cases of severe sepsis with known etiology successfully treated with ampicillin. The first patient was diagnosed with enterococcal spondylodiscitis related to colon diverticulitis. The second patient was a pregnant woman who developed listeriosis. The third patient was diagnosed with pneumococcal meningitis and the forth patient was diagnosed with bacteremic pneumococcal pneumonia. We chose ampicillin because of its narrow antimicrobial spectrum, among therapeutic alternatives. A cost efficiency analysis was performed for each patient.

## Conclusion

We emphasize the importance of microbiological diagnosis in order to use de-escalation. Ampicillin remains a very good option in severe infections produced by sensitive germs.

